# Supplementary material for: A massively parallel screening platform for converting aptamers into molecular switches
Source: Nat Commun. 2023 Apr 24;14:2336. doi: 10.1038/s41467-023-38105-4 (PMC10126150; doi:10.1038/s41467-023-38105-4)
Supplement: Supplementary file 1 — Supplementary Information [file 41467_2023_38105_MOESM1_ESM.pdf]

Supplementary Information:

Supplementary Tables:

Name	Sequence
ADS library	TCGTCGGCAGCGTCAGATGTGTATAAGAGACAG-T(25)-N(10)-TCTCAG CTGTCTCTTATACACATCTCCGAGCCCACGAGAC
ADS complement strand	GTCTCGTGGGCTCGGAGATGTGTATAAGAGACAGCTGAGA
ATP aptamer	QCACCTGGGGGAGTATTGCGGAGGAAGGTTTCTGTCTCTTATACACATCT GACGCTGCCGACGA
NNGmin glucose aptamer	QCCCGZGZZAGAZAGZAAGZGCAAZCZCGGCTTTCTGTCTCTTATACACAT CTGACGCTGCCGACGA
Ddel Test strand	Biotin-T(20)-N(10) – TCTCAGCTGTCTCTTATACACATCTCCGAGCCCACGAGAC-Cy5
FM	TCGTCGGCAGCGTCAGATGTGTATAAGAGACAGAACCAGGAGCAGGCCA AAAAGCGCGCAAGAATTCCTGTCTCTTATACACATCTCCGAGCCCACGAG AC
FM 532 comp	/5Alex532N/TTGCGCGCTTTTTGGCCTGCTCCTGGTT
FM 660 comp	/5Alex660N/TTGCGCGCTTTTTGGCCTGCTCCTGGTT

Supplementary Table 1: Sequences used in the switch screen. Z represents a boronic acid-modified U base. Q represents the Iowa Black FQ quencher.

Name	Sequence
atp-1	TCGTCGGCAGCGTCAGATGTGTATAAGAGACAGTTTTTTTTTTTTTTTTTT TTTTTTTCGACGCGTAATC-[Cy3]
atp-2	TCGTCGGCAGCGTCAGATGTGTATAAGAGACAGTTTTTTTTTTTTTTTTTT TTTTTTTACTCCAGCGATC-[Cy3]
atp-3	TCGTCGGCAGCGTCAGATGTGTATAAGAGACAGTTTTTTTTTTTTTTTTTT TTTTTTTCGTAGCTCACTC-[Cy3]
atp-4	TCGTCGGCAGCGTCAGATGTGTATAAGAGACAGTTTTTTTTTTTTTTTTTT TTTTTTTCGCATCCAGGTC-[Cy3]
atp-5	TCGTCGGCAGCGTCAGATGTGTATAAGAGACAGTTTTTTTTTTTTTTTTTT TTTTTTTGAGCAATATATC-[Cy3]
atp-6	TCGTCGGCAGCGTCAGATGTGTATAAGAGACAGTTTTTTTTTTTTTTTTTT TTTTTTTATGCGCTACTC-[Cy3]
atp-7	TCGTCGGCAGCGTCAGATGTGTATAAGAGACAGTTTTTTTTTTTTTTTTTT TTTTTTTCCGGGGGCTATC-[Cy3]
atp-8	TCGTCGGCAGCGTCAGATGTGTATAAGAGACAGTTTTTTTTTTTTTTTTTT TTTTTTTCCGGGGGCGATC-[Cy3]
atp1-s1	TCGTCGGCAGCGTCAGATGTGTATAAGAGACAGTTTTTTTTTTTTTTTTTT TTTTTTTGATCTACACCGG-[Cy3]
atp1-s2	TCGTCGGCAGCGTCAGATGTGTATAAGAGACAGTTTTTTTTTTTTTTTTTT TTTTTTTTACGGCCCATGA-[Cy3]
atp2-s1	TCGTCGGCAGCGTCAGATGTGTATAAGAGACAGTTTTTTTTTTTTTTTTTT TTTTTTTTACGCCAACCGT-[Cy3]
atp4-s1	TCGTCGGCAGCGTCAGATGTGTATAAGAGACAGTTTTTTTTTTTTTTTTTT TTTTTTTCCGTAGCGCCAT-[Cy3]
atp6-s1	TCGTCGGCAGCGTCAGATGTGTATAAGAGACAGTTTTTTTTTTTTTTTTTT TTTTTTTCCCTACTAGGT-[Cy3]

Supplementary Table 2: ATP switching strands and scrambled controls

Name	Sequence
GluPD library	GCGCATACCAGCTTATTCAATTNNNNNNNNNNNNNNNNNNNNNNNNNNNNNNNNNNNNNNNNNNNNNNNNNAGATAGTAAGTGCAATCTCGGC
Glu FP	GCGCATACCAGCTTATTCAATT
Glu RP	GCCGAGATTGCACTTACTATCT
Amino-PEG Glu FP	/5AmMC6//iSp18/GCGCATACCAGCTTATTCAATT
AF647-Glu RP	/5Alex647N/AATTGAATAAGCTGGTATGCGC
Bio-Glu RP	/5Biosg/GCCGAGATTGCACTTACTATCT
AF647 Glu FPC	/5Alex647N/AATTGAATAAGCTGGTATGCGC
NNG	GCGCAZZACCAGCZZAZZCAAZZGZAZCCZZAGZCCCAAZGZCZCCZGGCAACZCGCGCAGZGCCCCGZGZZAGAZAGZAAGZGCAAZCZCGGC
NNGmin	CCCGZGZZAGAZAGZAAGZGCAAZCZCGGC
Bio-NNGmin	/5BiotinTEG/CCCGZGZZAGAZAGZAAGZGCAAZCZCGGC

Supplementary Table 3: Sequences used in glucose aptamer selection and characterization. Z represents a boronic acid-modified U base.

Name	Sequence
glu-1	TCGTCGGCAGCGTCAGATGTGTATAAGAGACAGTTTTTTTTTTTTTTTTTT TTTTTTTAGGGCAGGCTC-[Cy3]
glu-2	TCGTCGGCAGCGTCAGATGTGTATAAGAGACAGTTTTTTTTTTTTTTTTTT TTTTTTCAGGACAGGCTC-[Cy3]
glu-3	TCGTCGGCAGCGTCAGATGTGTATAAGAGACAGTTTTTTTTTTTTTTTTTT TTTTTTTACCAGGAGCTC-[Cy3]
glu-4	TCGTCGGCAGCGTCAGATGTGTATAAGAGACAGTTTTTTTTTTTTTTTTTT TTTTTTACCAGGAGCTTC-[Cy3]
NNGmin	QCCGZGZZAGZAGZAAZGCAZCZCGGCTTTCTGTCTCTTATACACATCTGA CGCTGCCGACG
NNGmin _scr	QZGGZGCGCGCAGGCZCCZZAAZGCAZAAZTTTCTGTCTCTTATACACATC TGACGCTGCCGACG
NatGmin	QCCCGTGTTAGATAGTAAGTGCAATCTCGGCTTTCTGTCTCTTATACACAT CTGACGCTGCCGACG
glu1-s1	TCGTCGGCAGCGTCAGATGTGTATAAGAGACAGTTTTTTTTTTTTTTTTTT TTTTTTGGTAGAGCGCTC-[Cy3]
glu3-s1	TCGTCGGCAGCGTCAGATGTGTATAAGAGACAGTTTTTTTTTTTTTTTTTT TTTTTTACGCGATGCATC-[Cy3]
glu4-s1	TCGTCGGCAGCGTCAGATGTGTATAAGAGACAGTTTTTTTTTTTTTTTTTT TTTTTTGCGGATAACCTC-[Cy3]

Supplementary Table 4: Glucose switching strands and scrambled control sequences. Q represents the Iowa Black FQ quencher and Z represents a boronic acid-modified U base.

Time (min)	Flow (mL/min)	%A	%B
0	0.2	98	2
2	0.2	98	2
6	0.2	5	95
8	0.2	5	95
8.5	0.2	98	2

Supplementary Table 5: Chromatography gradient conditions for high resolution mass spectrometry (HRMS) analysis of Alexa Fluor 647 alkyne, Sulfo Cy5 Alkyne, and Alexa Fluor 647 conjugated glucose. A: 0.1% formic acid in HPLC grade water and B: 0.1% formic acid in acetonitrile.

Time (min)	Flow (mL/min)	%A	%B
0	1	95	5
2	1	95	5
15	1	70	30
20	1	3	97
24	1	3	97
26	1	95	5
34	1	95	5

Supplementary Table 6: Chromatography gradient conditions for HPLC analysis of Alexa Fluor 647 alkyne, and Sulfo Cy5 Alkyne. A: HPLC grade water and B: acetonitrile.

Supplementary Figures

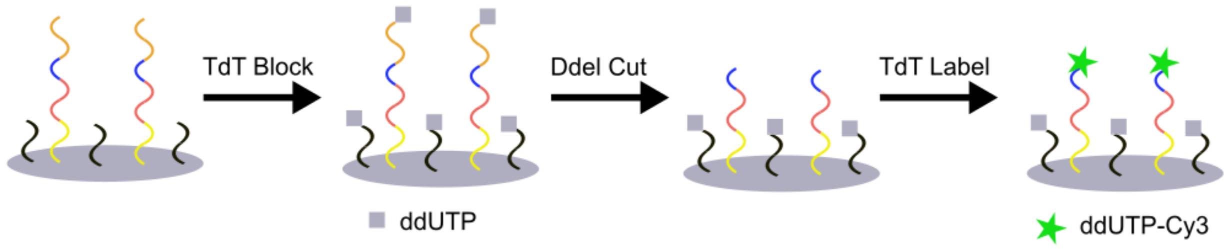

Supplementary Figure 1: Creation of fluorescently-tagged ADS clusters after MiSeq sequencing. First, the DNA clusters and sequencing primers are blocked using TdT enzyme with unlabeled ddUTP. Next, Ddel restriction enzyme is used to cleave the RP complement region from the ADS clusters, which also removes unlabeled ddUTP. Finally, the ADS clusters are fluorescently labeled using TdT in conjunction with ddUTP-cy3.

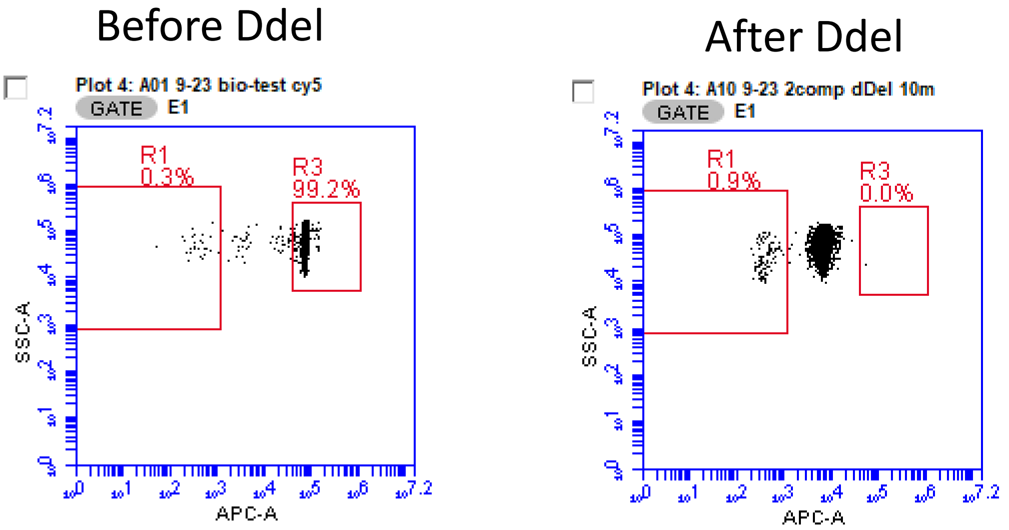

Supplementary Figure 2: Bead-based proof-of-concept experiment for restriction enzyme cleavage by Ddel. A 5'-biotinylated test strand was 3'-labeled with Cy3 and captured onto streptavidin-functionalized magnetic beads. A complementary strand was then annealed on, and the beads were subjected to Ddel enzymatic cleavage. The beads were analyzed via flow-cytometry before and after the enzymatic cleavage. The decrease in fluorescence after the addition of the Ddel indicates successful cleavage of the DNA. See the **Methods** section ("Validation of TdT and Ddel enzymes on beads") for additional details.

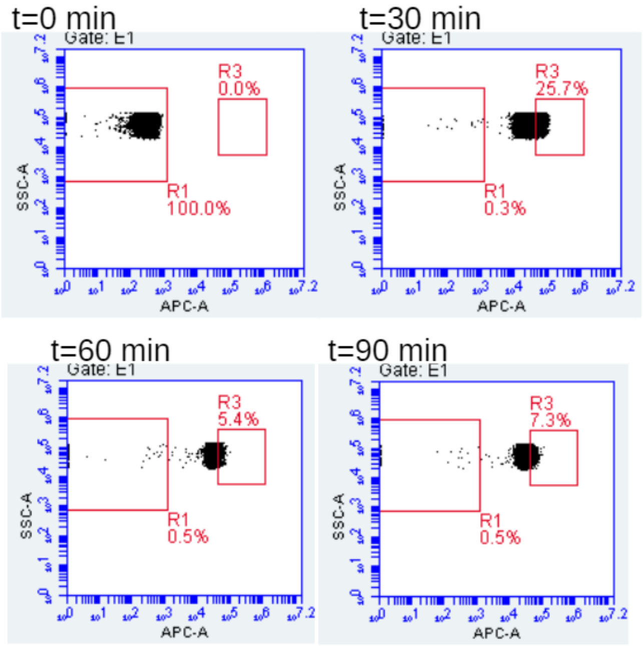

Supplementary Figure 3: Bead-based proof-of-concept experiment for TdT ligation of ddUTP. The increase in fluorescence indicates successful incorporation of fluorescently-labeled ddUTP by TdT.

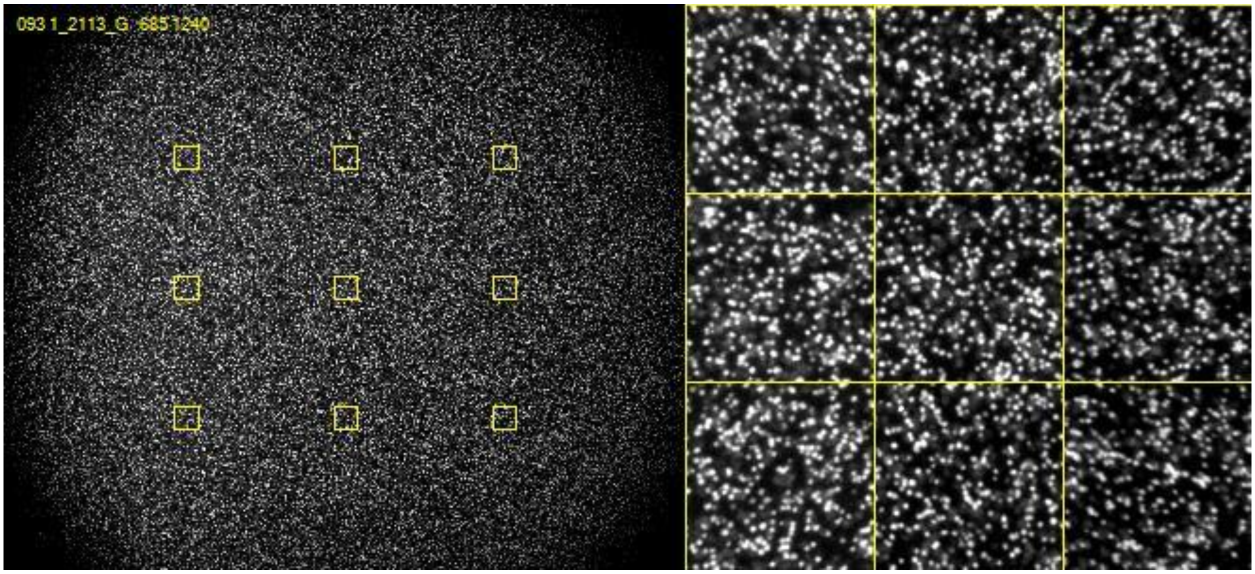

Supplementary Figure 4: MiSeq flow-cell tile after TdT blocking, DdeI cutting, and TdT labeling steps. The image is from the G channel, which can detect the Cy3 fluorophore. This experiment was performed a single time.

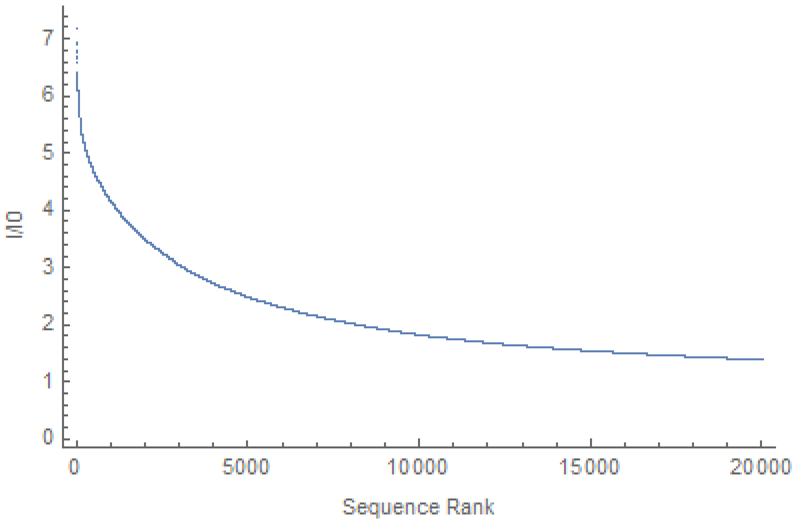

Supplementary Figure 5: The ratio of intensity after target addition to initial intensity in buffer for the ATP switch selection for the top 20,000 signal-on sequences. Source data are provided as a Source Data file.

DISCOVERED MOTIFS

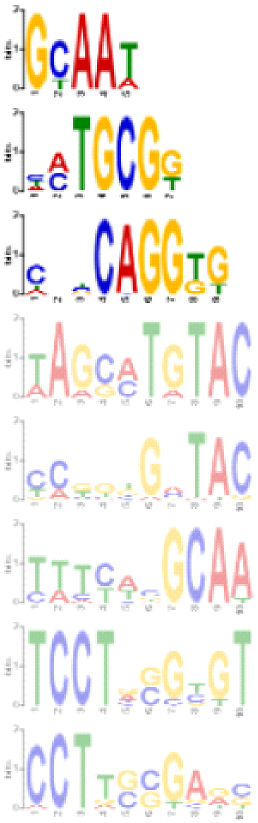

	Logo	E-value	Sites	Width	More	Submit/Download
1.		1.6e-037	282	5		
2.		5.0e-008	93	7		
3.		1.5e-006	49	9		
4.		4.7e-001	7	10		
5.		2.3e+000	32	10		
6.		2.0e+001	13	10		
7.		4.9e+001	8	10		
8.		1.3e+001	18	10		

Stopped because requested number of motifs (8) found.

Supplementary Figure 6: Output after running the MEME motif discovery program[1] on the top 1,000 unique signal-on ATP switch sequences. Classic mode was used with default settings, and the algorithm was limited to eight total motifs. Only the first three motifs were considered statistically significant based on E-values > 0.05.

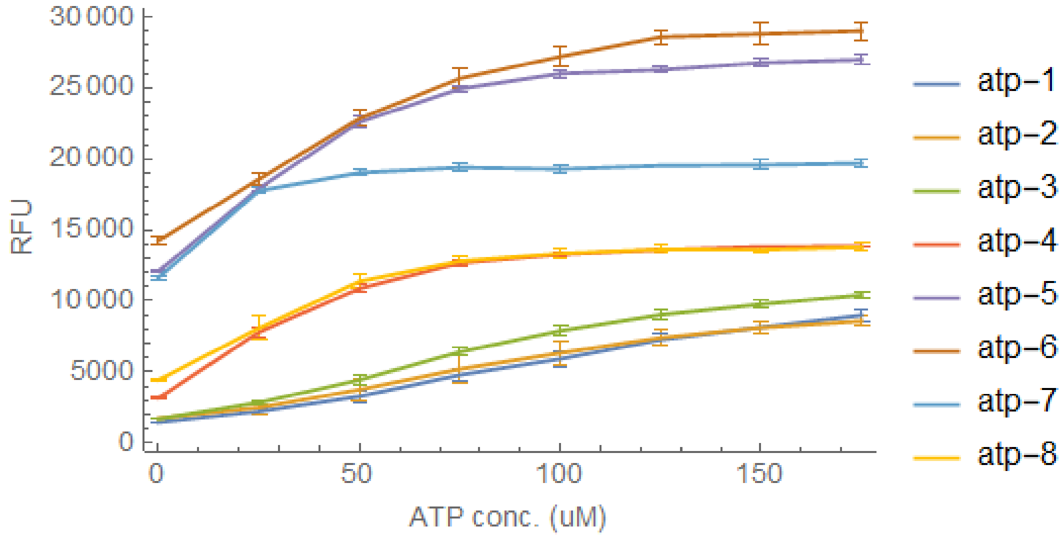

Supplementary Figure 7: Raw RFU values from ATP switch plate-reader binding experiments. Each point represents the mean of quadruplicate experiments, and the error bars represent the standard deviation. Source data are provided as a Source Data file.

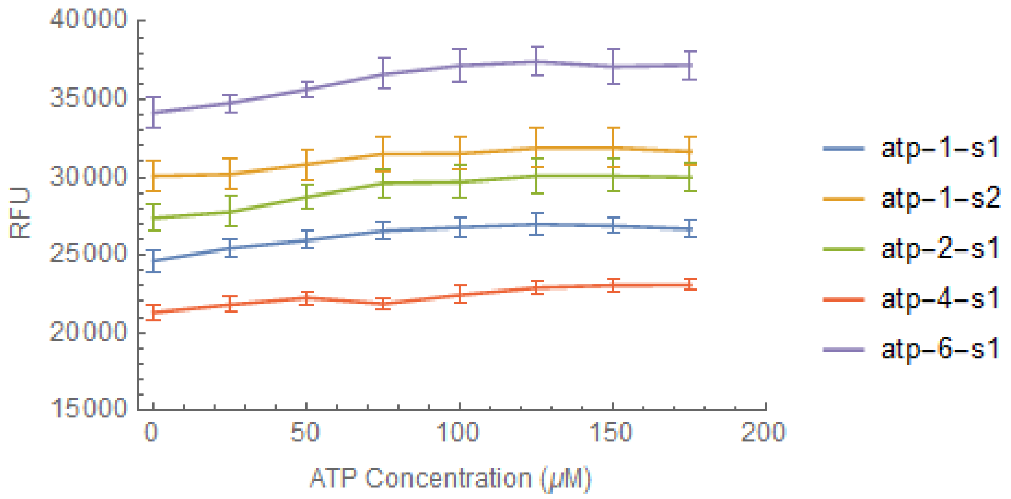

Supplementary Figure 8: Raw RFU values from plate-reader binding experiments with scrambled versions of the atp-1, atp-2, atp-4, and atp-6 sequences. Each point represents the mean of quadruplicate experiments, and the error bars represent the standard deviation. Source data are provided as a Source Data file.

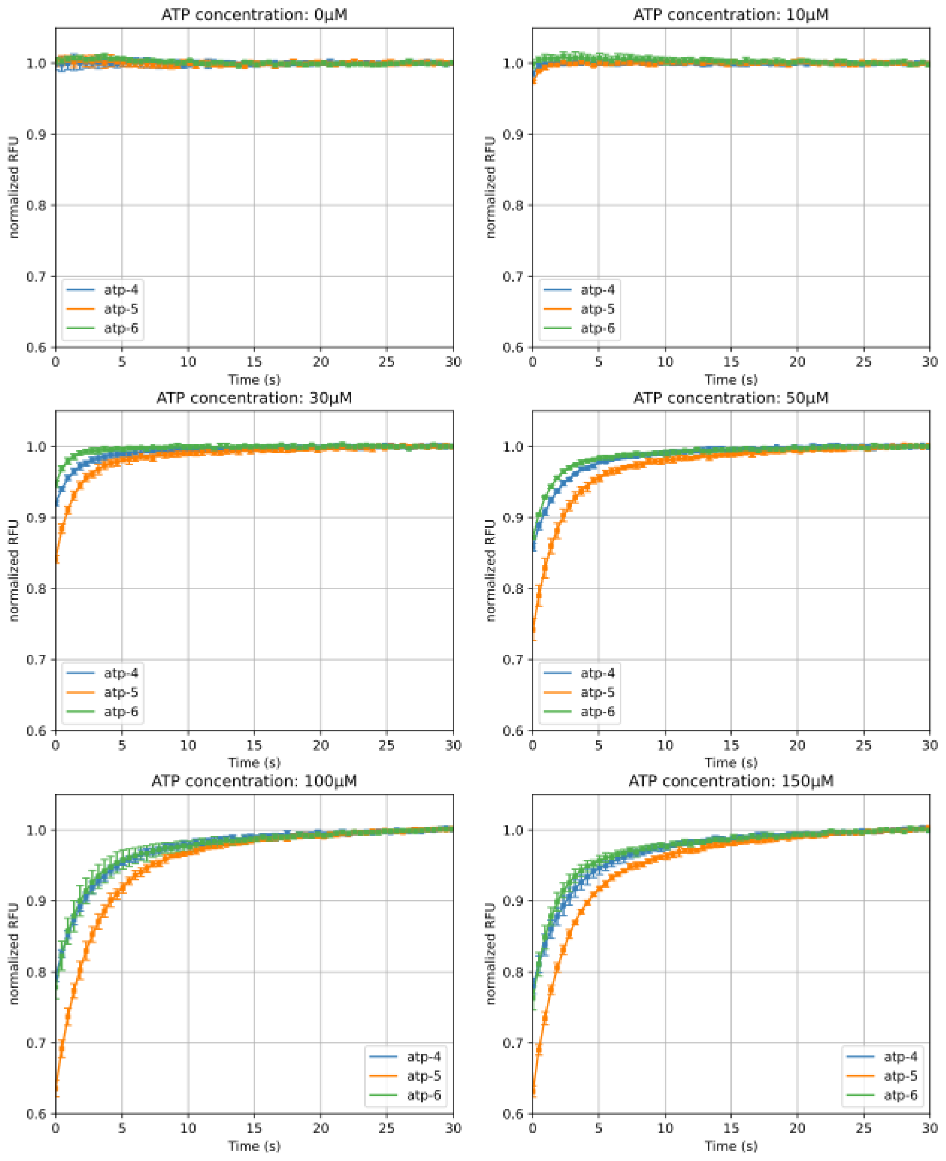

Supplementary Figure 9: Kinetic binding analysis of atp-4, atp-5, and atp-6 switches. Kinetic binding behavior was examined at various concentrations of ATP. Signals are normalized to the final RFU values. Each data point represents the mean of three independent experiments and the error bars represent a single standard deviation. Source data are provided as a Source Data file.

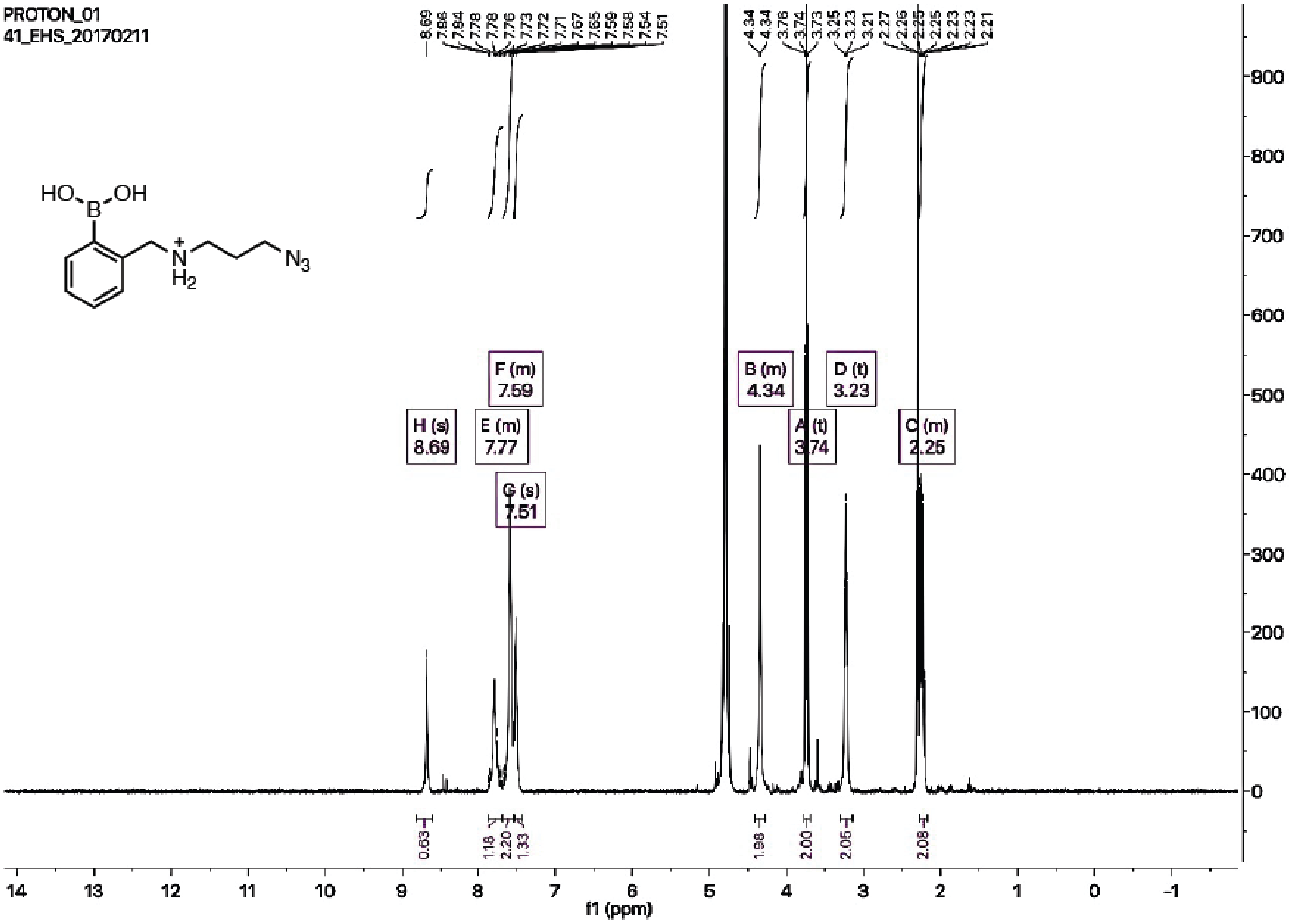

Supplementary Figure 10: ^{1}H-NMR characterization of azido phenylboronic acid.

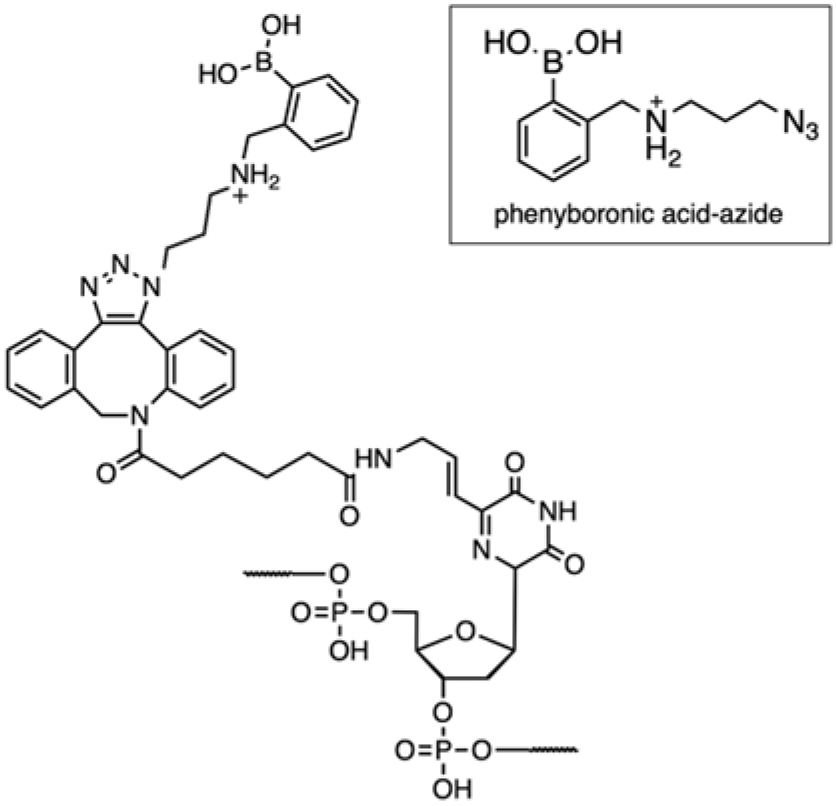

Supplementary Figure 11: Chemical structure of the boronic acid-modified dU used in our glucose switch constructs. The modified base is generated through a two-step conjugation of an aminoallyl dU to DBCO-NHS ester followed by phenylboronic acid-azide.

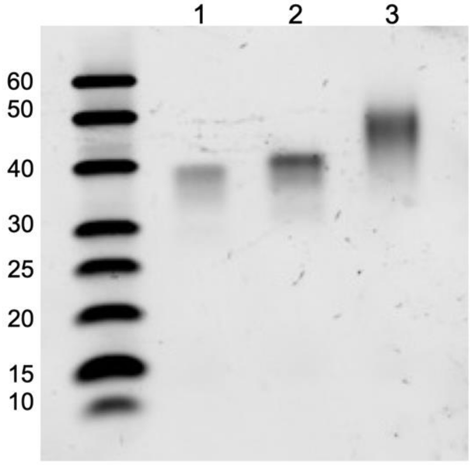

Supplementary Figure 12: Validation of click chemistry yield using denaturing PAGE. Gel lanes represent 1) Bio-NNGmin 2) Bio-NNGmin after reaction with DBCO-NHS ester, and 3) Bio-NNGmin after reaction with both DBCO NHS ester and phenylboronic acid azide. The leftmost lane is a 10/60 single-stranded DNA oligo length standard (IDT Catalog #51-05-15-01) and

contains marker oligos of 10, 15, 20, 25, 30, 40, 50, 60, 70, 80, 90, and 100 bases. The length of each reference oligo is labeled. The experiment was repeated twice (n = 2) with similar results. Uncropped gel image is provided in Source Data file.

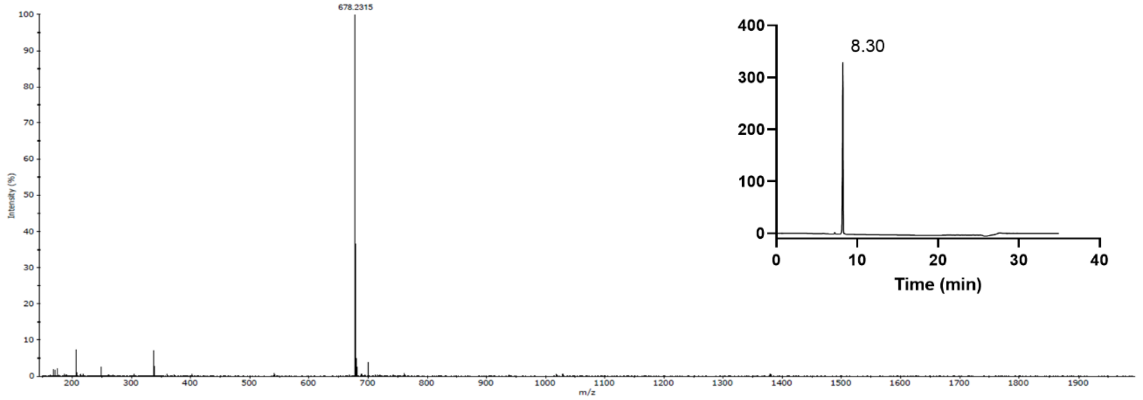

Supplementary Figure 13: HRMS spectrum of AlexaFluor 647 alkyne, inset: chromatogram of AlexaFluor 647 alkyne. [$C_{35}H_{41}N_3O_7S_2$-H^+] calculated m/z = 678.2313, observed m/z = 678.2315.

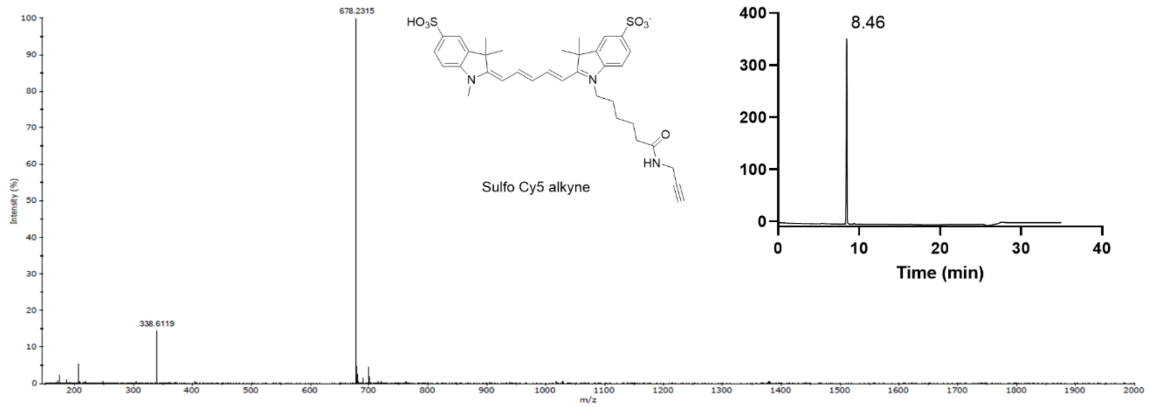

Supplementary Figure 14: HRMS spectrum of sulfo Cy5 alkyne, inset: chromatogram of sulfo Cy5 alkyne. [$C_{35}H_{41}N_3O_7S_2$-H^+] calculated m/z = 678.2313, observed m/z = 678.2315.

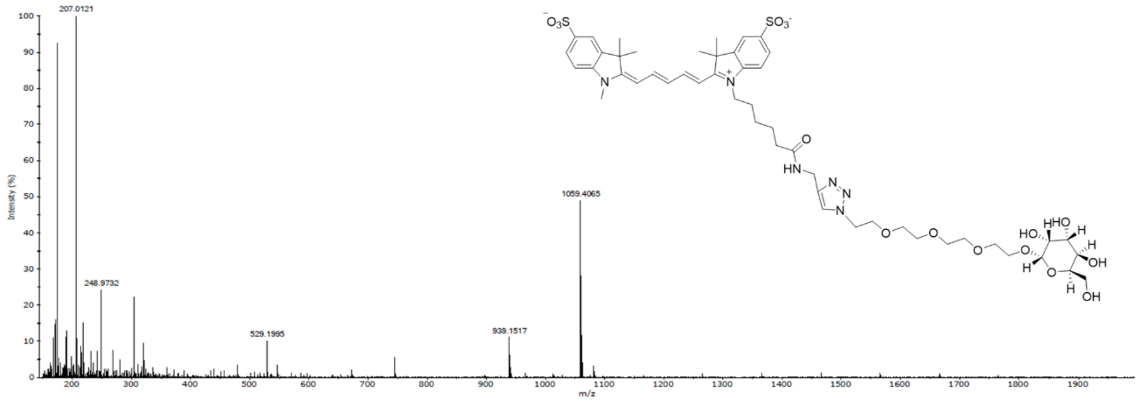

Supplementary Figure 15: HRMS spectrum of AlexaFluor 647-labeled glucose. [$C_{49}H_{68}N_6O_{16}S_2$-H^+] calculated m/z = 1059.4060, observed m/z = 1059.4065.

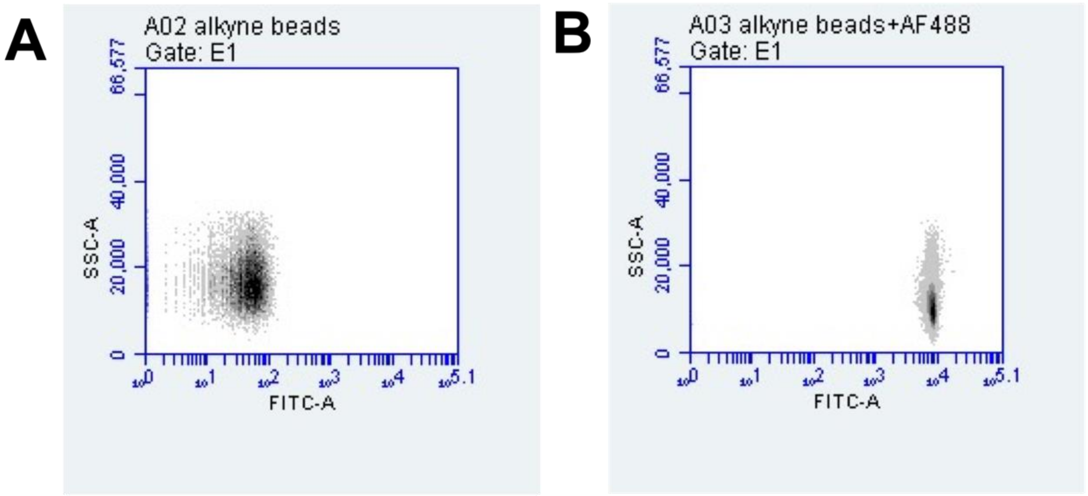

Supplementary Figure 16: Flow cytometry validation of conjugation to alkyne magnetic beads during pre-enrichment. Beads were monitored in the FITC channel before and after reaction with azide-modified AlexaFluor 488 using CuAAC click chemistry to assess the efficiency of conjugation to bead surfaces.

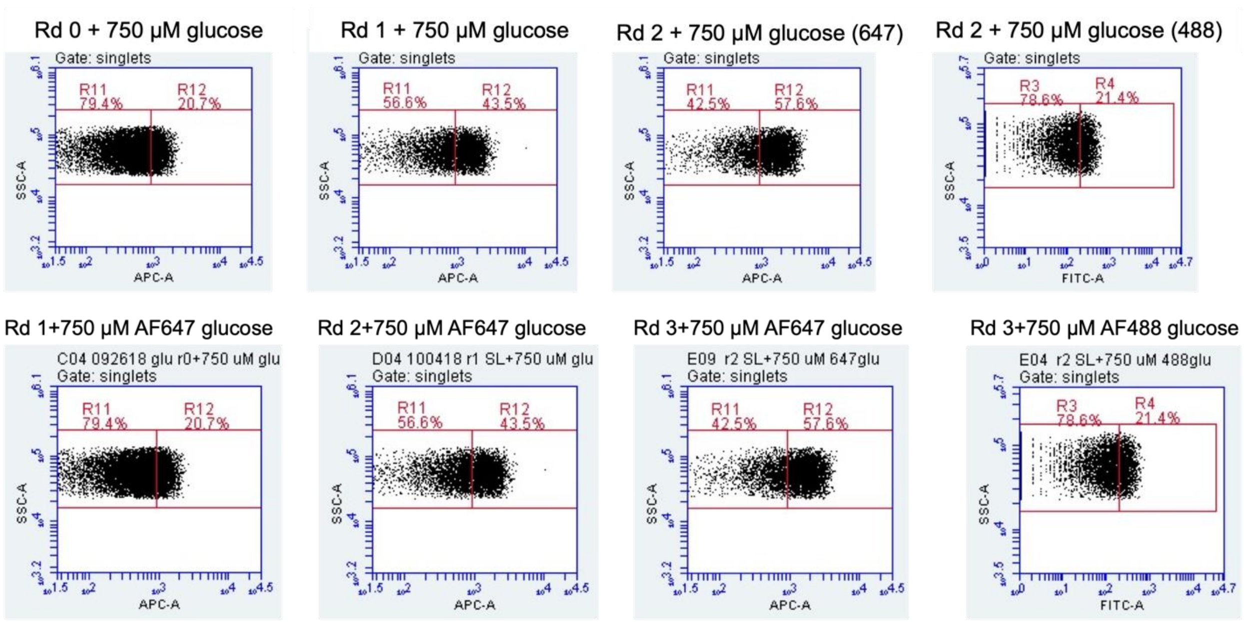

Supplementary Figure 17: Round to round monitoring of enrichment of aptamer pools during particle display. The aptamer pool was incubated with AF647-labeled glucose for two rounds and monitored in the APC channel. In round 3, the library was incubated with both AF647 and 488-labeled glucose and monitored in the APC and FITC channel respectively. AF488 was used in the third round of PD sorting to discourage and sequences that bind to the AF647 fluorophore. The particles were sorted with 750 μM glucose in all three rounds.

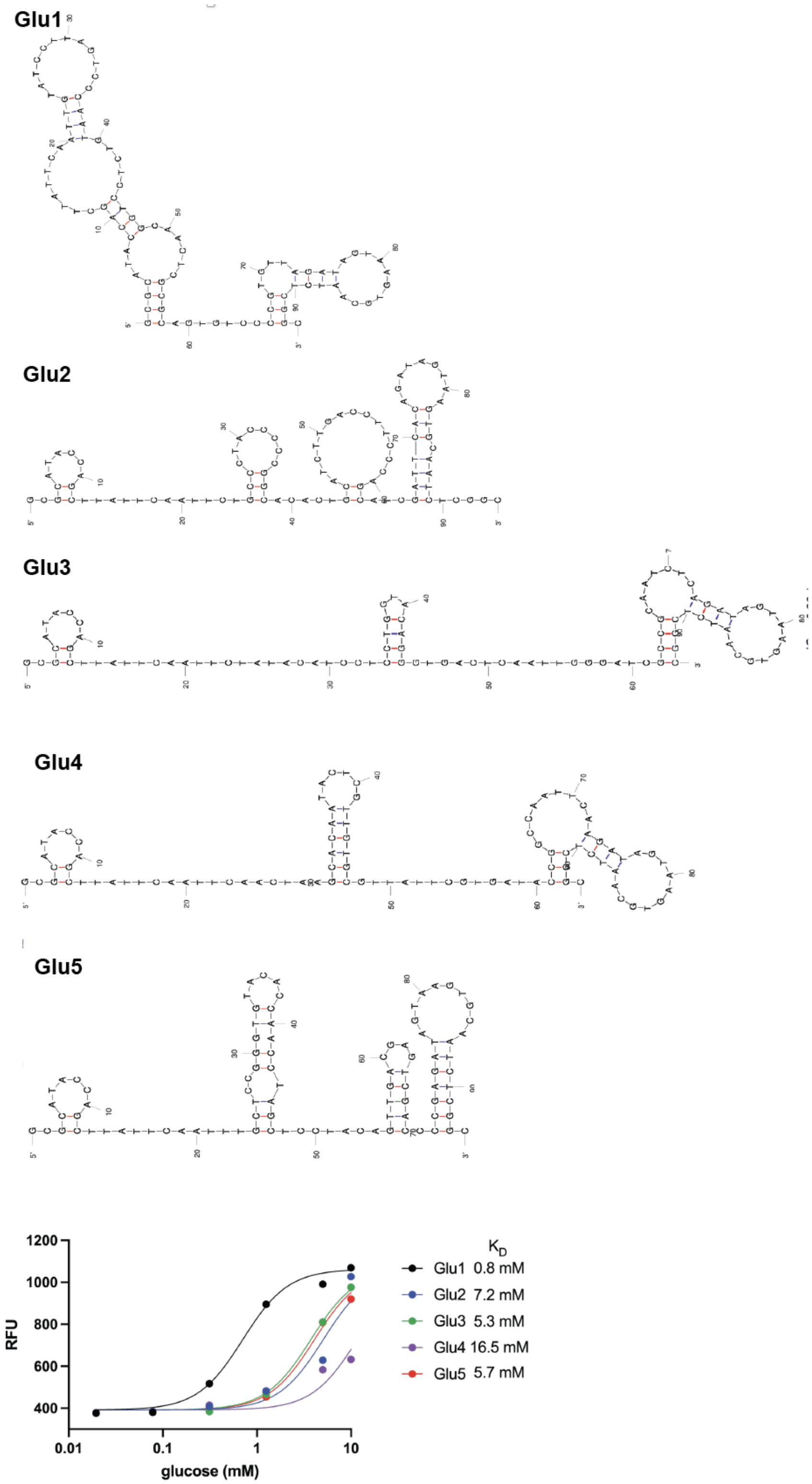

Supplementary Figure 18: Secondary structure prediction for the five highest copy-number candidate sequences from the PD screen (top) and initial binding validation using flow cytometry (bottom). Source data are provided as a Source Data file.

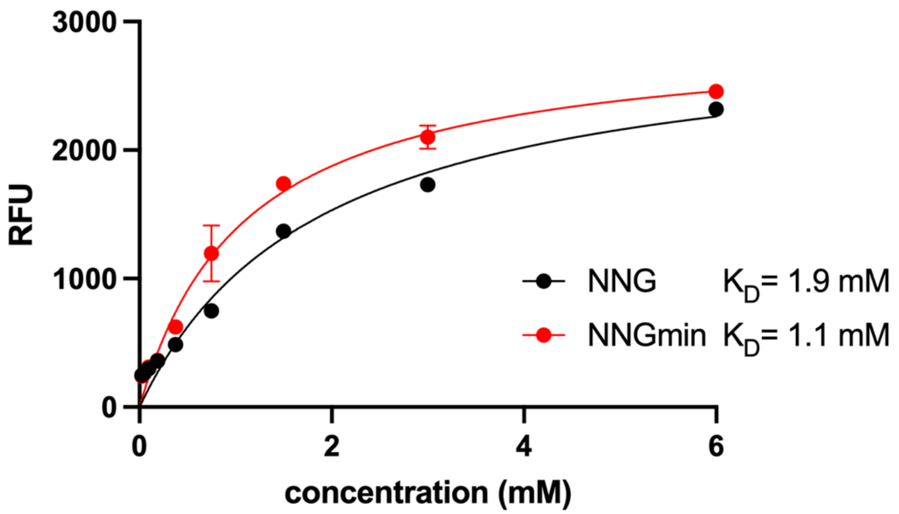

Supplementary Figure 19: Flow cytometry-based binding assay of full-length NNG and minimized glucose aptamer NNGmin. Each point represents the mean (n=3), and the error bars represent a single standard deviation. Source data are provided as a Source Data file.

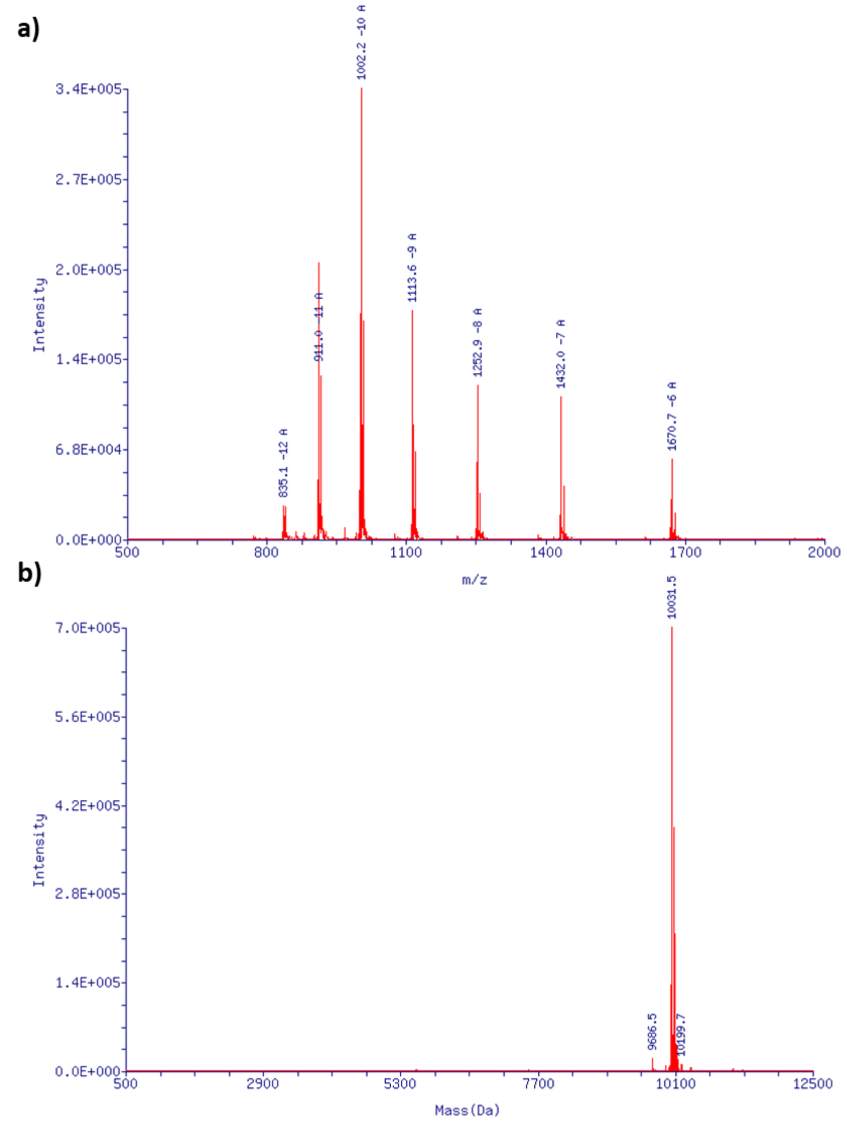

Supplementary Figure 20: **a**) ESI-MS spectrum of Bio-NNGmin and **b**) deconvoluted ESI-MS spectrum of Bio-NNGmin. Calculated mass: 10,026.71; observed mass: 10,031.5.

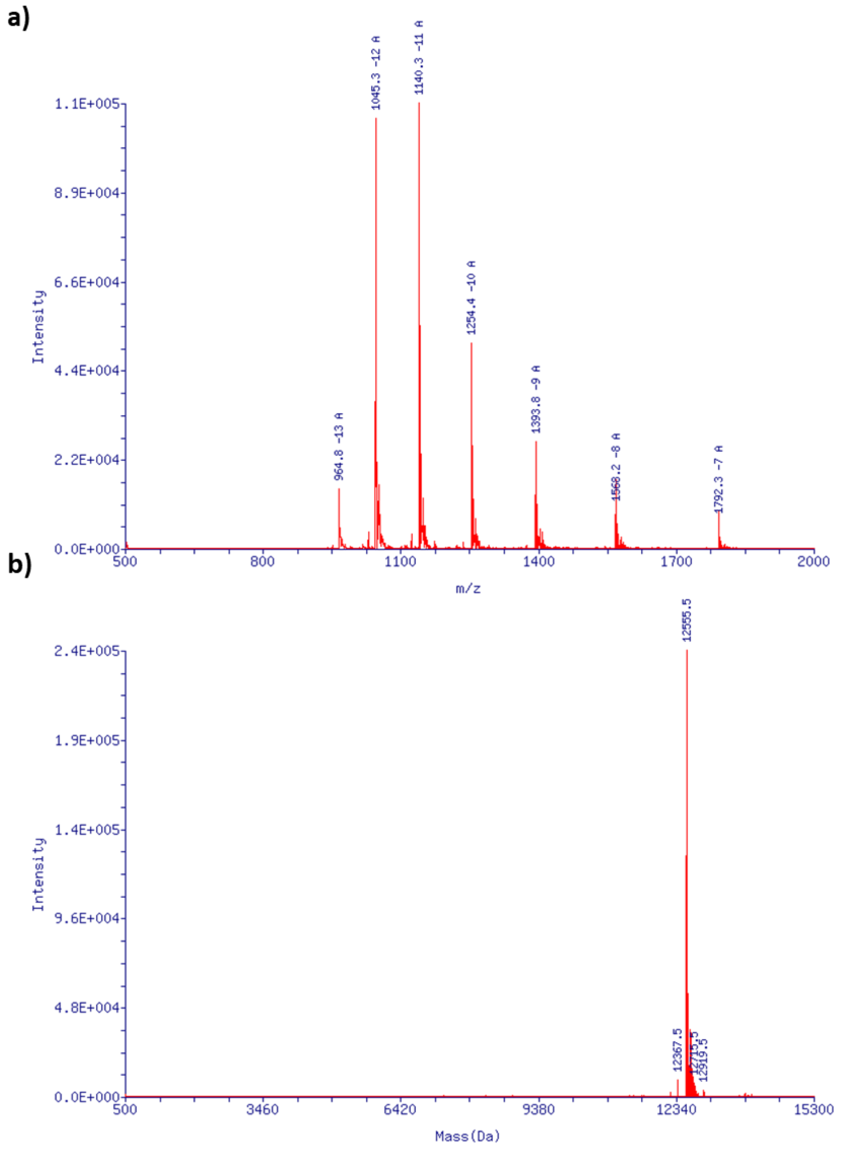

Supplementary Figure 21: a) ESI-MS spectrum of Bio-NNGmin after reaction with DBCO sulfo-NHS ester (BioNNGmin+DBCO), and **b**) deconvoluted ESI-MS spectrum of BioNNGmin+DBCO. Calculated mass: 12,561.46; observed mass: 12,555.5.

.

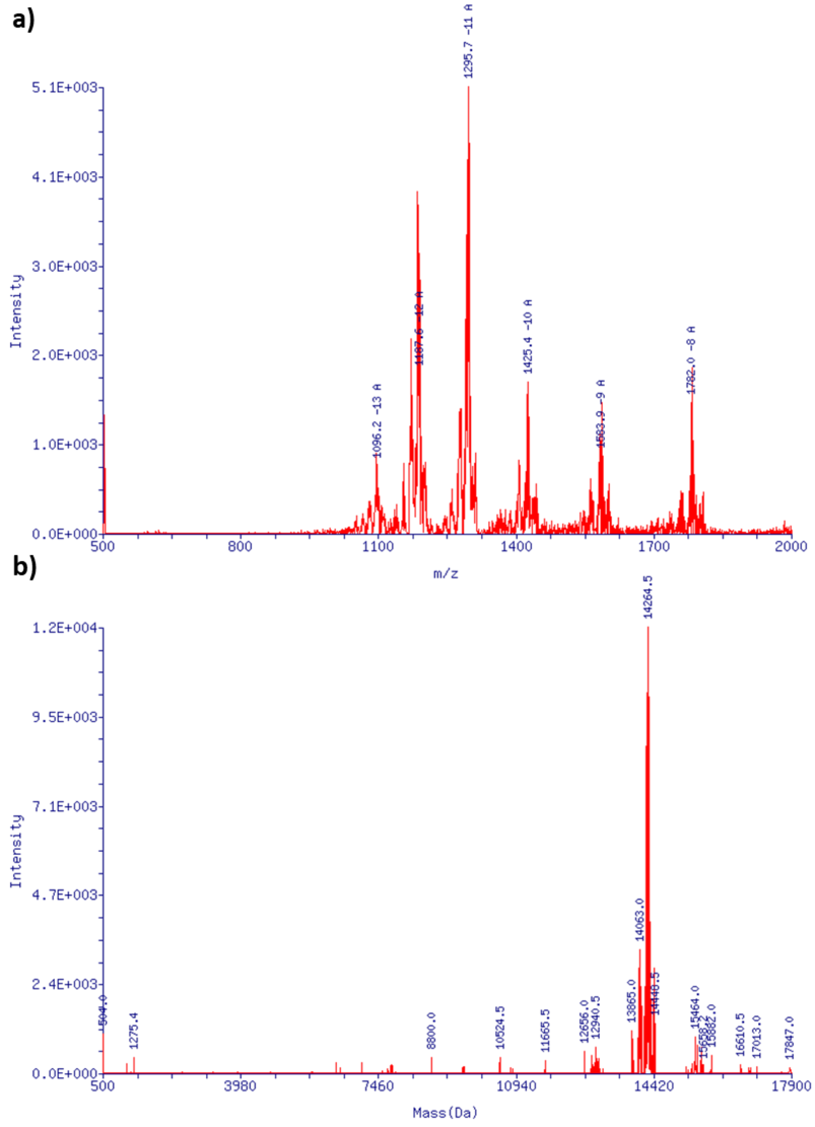

Supplementary Figure 22: a) ESI-MS spectrum of Bio-NNGmin+BA, and **b**) deconvoluted ESI-MS spectrum of Bio-NNGmin+BA. Calculated mass: 14,442.6 (eight DBCO groups conjugated), 14,207.5 (seven DBCO groups conjugated); observed mass: 14,264.5 (M-BA-3H+Na+K, calculated mass 14,266.5).

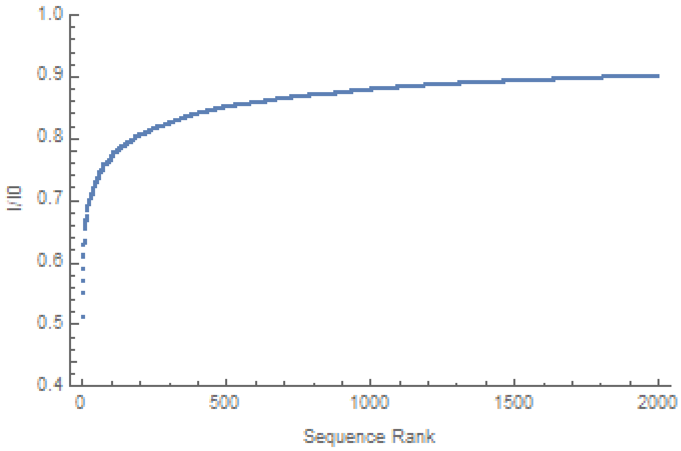

Supplementary Figure 23: The ratio of intensity after target addition to initial intensity in buffer for the top 2,000 signal-off sequences in the glucose switch selection. Source data are provided as a Source Data file.

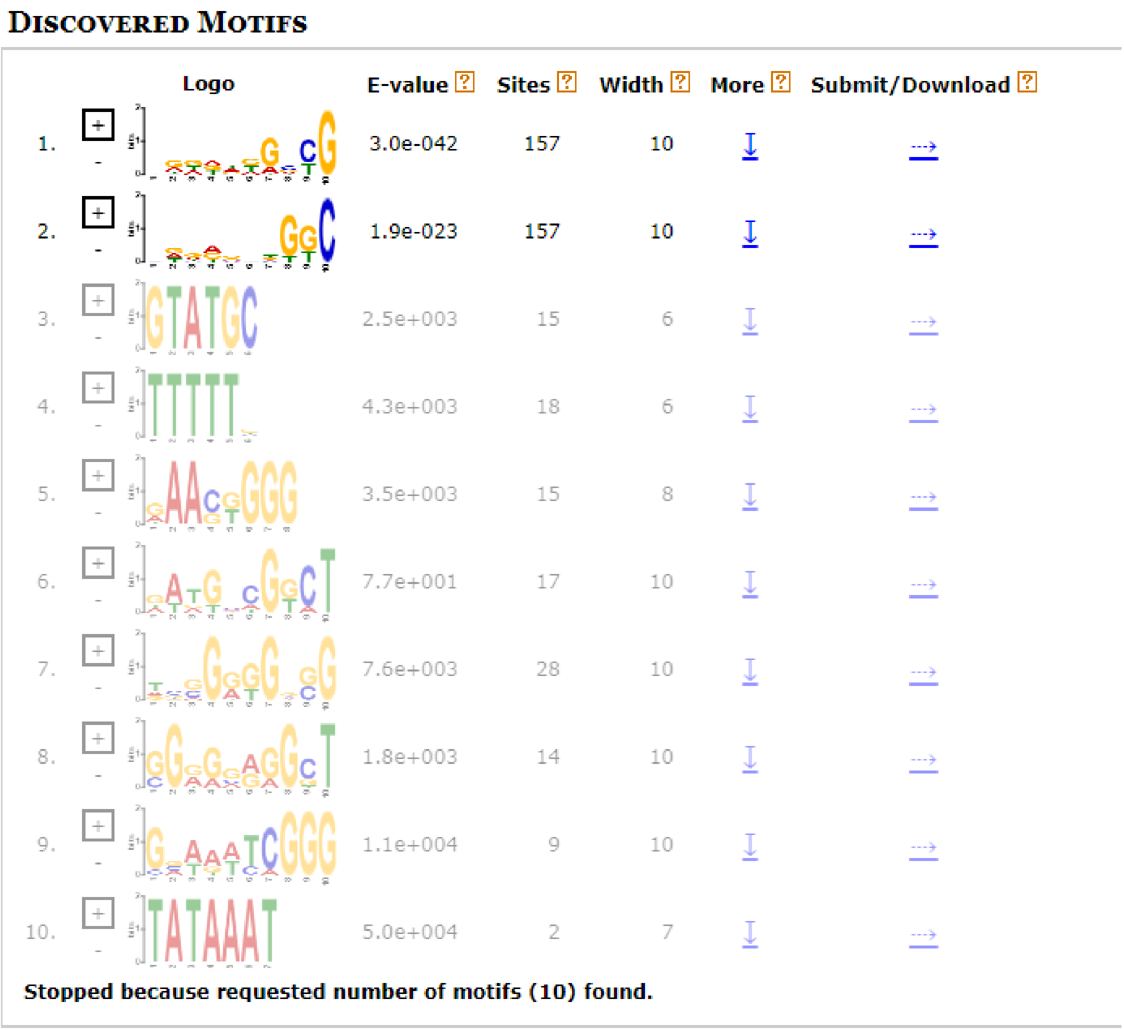

Supplementary Figure 24: Output for the MEME motif discovery program[1] from the top 1,000 unique signal-off sequences for glucose. Classic mode was used with default settings, and the algorithm was limited to 10 total motifs. Only the first three motifs were considered statistically significant based on E-values >0.05.

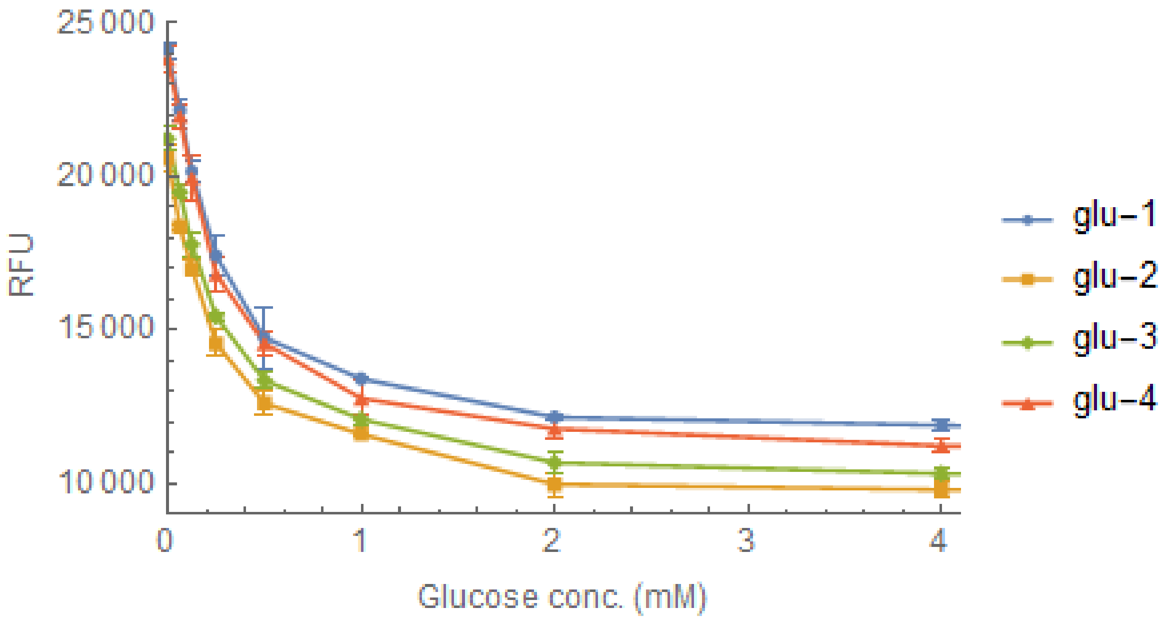

Supplementary Figure 25: Raw RFU values from glucose switch plate-reader validation experiments. Each data point represents the mean value of three independent experiments, and the error bars represent the standard deviation of the measurements. Source data are provided as a Source Data file.

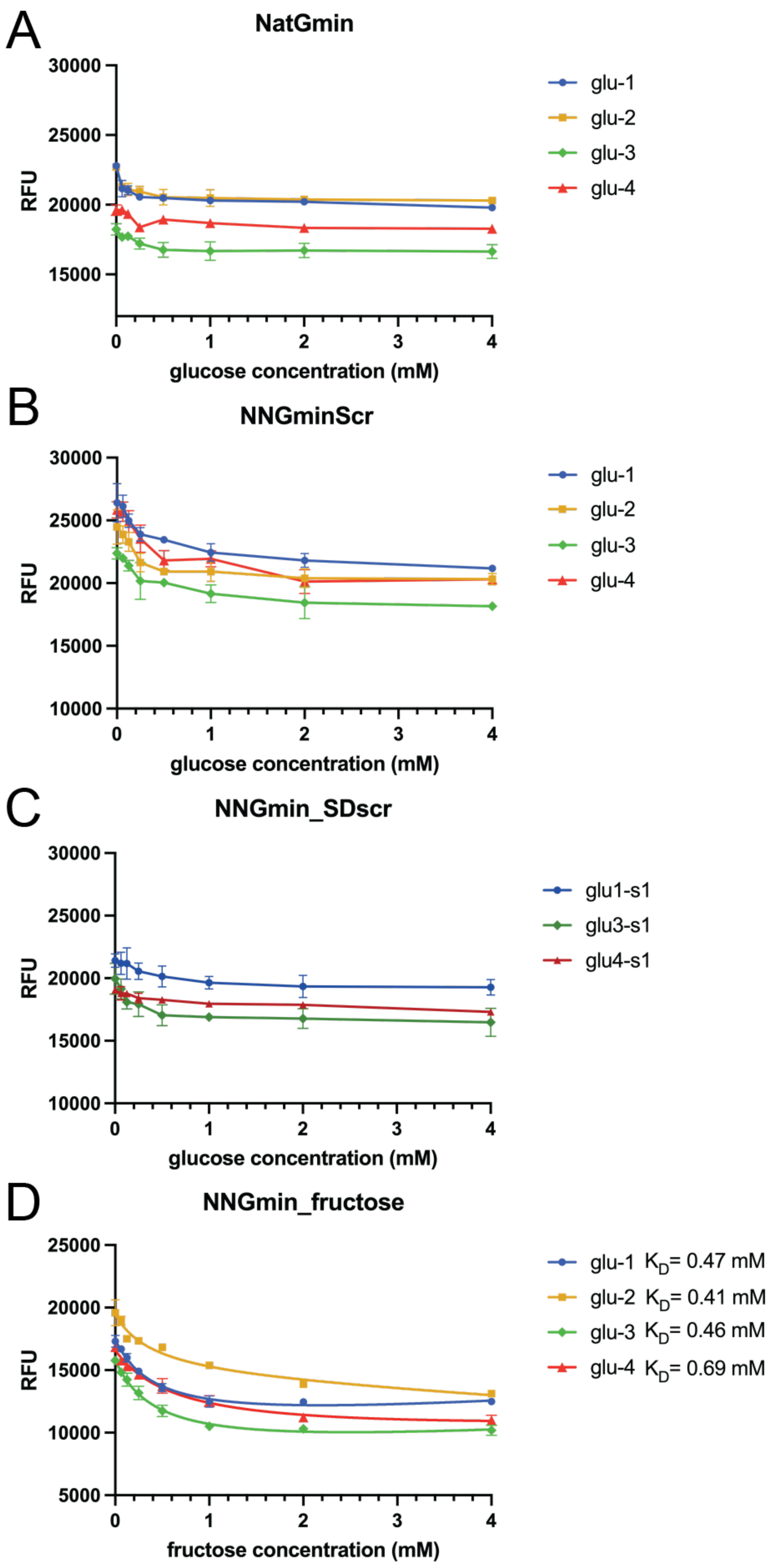

A
NatGmin
RFU
30000
25000
20000
15000
0
1
2
3
4
glucose concentration (mM)
glu-1
glu-2
glu-3
glu-4
B
NNGminScr
RFU
30000
25000
20000
15000
10000
0
1
2
3
4
glucose concentration (mM)
glu-1
glu-2
glu-3
glu-4
C
NNGmin_SDscr
RFU
30000
25000
20000
15000
10000
0
1
2
3
4
glucose concentration (mM)
glu1-s1
glu3-s1
glu4-s1
D
NNGmin_fructose
RFU
25000
20000
15000
10000
5000
0
1
2
3
4
fructose concentration (mM)
glu-1 K_D= 0.47 mM
glu-2 K_D= 0.41 mM
glu-3 K_D= 0.46 mM
glu-4 K_D= 0.69 mM

Supplementary Figure 26: Plate reader validation for specificity control experiments. Raw RFU values for A) a natural DNA aptamer analog negative control (NatGmin) with switching strands glu1–4, B) a scrambled aptamer variant negative control (NNGminScr) with switch strands glu1–4, C) NNGmin with scrambled negative control SD sequences glu1/3/4s-1, and D) NNGmin with switching strands glu1–4 binding to fructose. Data-points are the mean of three independent experiments and the error bars represent a single standard deviation. Source data are provided as a Source Data file.

Supplementary References:

(1) Bailey, T. L.; Boden, M.; Buske, F. A.; Frith, M.; Grant, C. E.; Clementi, L.; Ren, J.; Li, W. W.; Noble, W. S. MEME Suite: Tools for Motif Discovery and Searching. *Nucleic Acids Research* **2009**, *37* (SUPPL. 2), 202–208. https://doi.org/10.1093/nar/gkp335.
