# Supplementary material for: A massively parallel screening platform for converting aptamers into molecular switches
Source: Nat Commun. 2023 Apr 24;14:2336. doi: 10.1038/s41467-023-38105-4 (PMC10126150; doi:10.1038/s41467-023-38105-4)
Supplement: Supplementary file 3 — Description of additional supplementary files [file 41467_2023_38105_MOESM3_ESM.pdf]

Description of additional supplementary files

Supplementary Dataset 1:

This dataset contains processed data files from the high-throughput ATP aptamer switch screen. The dataset includes the sequence of each cluster on the MiSeq flow cell, as well as the computed fluorescent intensity during each cycle of the screen.

Supplementary Dataset 2:

This dataset contains processed data files from the high-throughput glucose aptamer switch screen. The dataset includes the sequence of each cluster on the MiSeq flow cell, as well as the computed fluorescent intensity during each cycle of the screen.
