# Supplementary material for: A massively parallel screening platform for converting aptamers into molecular switches
Source: Nat Commun. 2023 Apr 24;14:2336. doi: 10.1038/s41467-023-38105-4 (PMC10126150; doi:10.1038/s41467-023-38105-4)
Supplement: Supplementary file 6 — Reporting Summary [file 41467_2023_38105_MOESM6_ESM.pdf]

nature portfolio

Corresponding author(s): H. Tom Soh

Last updated by author(s): Apr 6, 2023

Reporting Summary

Nature Portfolio wishes to improve the reproducibility of the work that we publish. This form provides structure for consistency and transparency in reporting. For further information on Nature Portfolio policies, see our Editorial Policies and the Editorial Policy Checklist.

Statistics

For all statistical analyses, confirm that the following items are present in the figure legend, table legend, main text, or Methods section.

n/a	Confirmed	
☐	☒	The exact sample size (*n*) for each experimental group/condition, given as a discrete number and unit of measurement
☐	☒	A statement on whether measurements were taken from distinct samples or whether the same sample was measured repeatedly
☒	☐	The statistical test(s) used AND whether they are one- or two-sided<br>*Only common tests should be described solely by name; describe more complex techniques in the Methods section.*
☒	☐	A description of all covariates tested
☒	☐	A description of any assumptions or corrections, such as tests of normality and adjustment for multiple comparisons
☐	☒	A full description of the statistical parameters including central tendency (e.g. means) or other basic estimates (e.g. regression coefficient) AND variation (e.g. standard deviation) or associated estimates of uncertainty (e.g. confidence intervals)
☒	☐	For null hypothesis testing, the test statistic (e.g. *F*, *t*, *r*) with confidence intervals, effect sizes, degrees of freedom and *P* value noted<br>*Give P values as exact values whenever suitable.*
☒	☐	For Bayesian analysis, information on the choice of priors and Markov chain Monte Carlo settings
☒	☐	For hierarchical and complex designs, identification of the appropriate level for tests and full reporting of outcomes
☒	☐	Estimates of effect sizes (e.g. Cohen's *d*, Pearson's *r*), indicating how they were calculated

Our web collection on statistics for biologists contains articles on many of the points above.

Software and code

Policy information about availability of computer code

Data collection	For sorting beads, BD FACSDiva software (version 8.0.2) was used. For experiments conducted with the Synergy H1 BioTek platereader, Gen5 software (version 3.04.17) was used. Folder agent version 2016 (123) and MiSeq Control software (version 2.6.2.1) were utilized for the high-throughput screen.
Data analysis	Wolfram Mathematica version 12 (version 12.0.0.0) was used to create bar charts, binding curves, fit curves, and calculate statistics. For extraction of the bead-based binding assay values, BD Accuri C6 Plus Software (version 1.0.23.1) was used.<br><br>The MiSeq data linking code for the platform is available on our GitHub account (https://github.com/sohlab/non-natural-aptamer-array), and the corresponding pseudocode is provided in the Methods section of the manuscript.

For manuscripts utilizing custom algorithms or software that are central to the research but not yet described in published literature, software must be made available to editors and reviewers. We strongly encourage code deposition in a community repository (e.g. GitHub). See the Nature Portfolio guidelines for submitting code & software for further information.

Data

Policy information about availability of data

All manuscripts must include a data availability statement. This statement should provide the following information, where applicable:

- Accession codes, unique identifiers, or web links for publicly available datasets
- A description of any restrictions on data availability
- For clinical datasets or third party data, please ensure that the statement adheres to our policy

The raw sequencing data generated during the aptamer switch screen experiments are available under NCBI Sequence Read Archive (SRA) (Accession code: PRJNA952942), which can be accessed at https://www.ncbi.nlm.nih.gov/sra/PRJNA952942. The data underlying Figures 2A, 2B, 3A, 3C, 4B, 4C, 5A, and 5C are contained in the Source Data File. The data underlying Supplementary Figures 5, 7, 8, 9, 12, 18, 19, 23, 25, and 26 are also contained in the Source Data File. The underlying processed data files from the high-throughput screen for both the ATP and glucose instrument runs are provided as supplementary data files. Any additional data from this study is available from the authors upon reasonable request. Source Data are provided with this paper.

Human research participants

Policy information about studies involving human research participants and Sex and Gender in Research.

Reporting on sex and gender	The manuscript refers to the in-vitro development of molecular sensors and does not refer to any human or animal subjects. The reporting on sex and gender is not relevant to this manuscript.
Population characteristics	Not relevant since our study does not contain human participants.
Recruitment	Not relevant since our study does not contain human participants.
Ethics oversight	Not relevant since our study does not contain human participants.

Note that full information on the approval of the study protocol must also be provided in the manuscript.

Field-specific reporting

Please select the one below that is the best fit for your research. If you are not sure, read the appropriate sections before making your selection.

☒ Life sciences ☐ Behavioural & social sciences ☐ Ecological, evolutionary & environmental sciences

For a reference copy of the document with all sections, see nature.com/documents/nr-reporting-summary-flat.pdf

Life sciences study design

All studies must disclose on these points even when the disclosure is negative.

Sample size	Triplicate measurement is the gold standard for the characterization of aptamer binding interactions and allows the determination of sample-to-sample variance. Triplicate measurements are utilized since it is the minimum sample number required to obtain a standard deviation which is necessary to identify potential outliers as well as compare the results of two experimental conditions in a statistically meaningful manner. We followed the conventional gold standard as a minimum threshold and conducted at least triplicate measurements for all experiments characterizing the aptamer reagents in this study.
Data exclusions	No data was excluded from this study.
Replication	All attempts to replicate results were success. The number of times each experiment was replicated is indicated in the caption for each figure containing the experimental data. For the high-throughput screening experiments and characterization of the aptamer reagents the results were replicated a minimum of three times.
Randomization	Each sample was tested under the same experimental conditions. The in-vitro tests were conducted in a well-controlled environment so no randomization was required.
Blinding	No blinding was required as the identity of each sample was known prior to each measurement.

Reporting for specific materials, systems and methods

We require information from authors about some types of materials, experimental systems and methods used in many studies. Here, indicate whether each material, system or method listed is relevant to your study. If you are not sure if a list item applies to your research, read the appropriate section before selecting a response.

Materials & experimental systems

n/a	Involved in the study
☒	☐ Antibodies
☒	☐ Eukaryotic cell lines
☒	☐ Palaeontology and archaeology
☒	☐ Animals and other organisms
☒	☐ Clinical data
☒	☐ Dual use research of concern

Methods

n/a	Involved in the study
☒	☐ ChIP-seq
☐	☒ Flow cytometry
☒	☐ MRI-based neuroimaging

Flow Cytometry

Plots

Confirm that:

☒ The axis labels state the marker and fluorochrome used (e.g. CD4-FITC).

☒ The axis scales are clearly visible. Include numbers along axes only for bottom left plot of group (a 'group' is an analysis of identical markers).

☒ All plots are contour plots with outliers or pseudocolor plots.

☒ A numerical value for number of cells or percentage (with statistics) is provided.

Methodology

Sample preparation	No cells or biological samples were used. Beads expressing aptamer sequences were created via emulsion PCR as outlined in the methods section.
Instrument	For sorting beads a BD FACSAria III Cell Sorter was used (model #648282-01).
Software	For sorting beads, BD FACSDiva software (version 8.0.2) was used. For analysis of bead-based binding assays, BD Accuri C6 Plus Software (version 1.0.23.1) was used.
Cell population abundance	This is not relevant since no cells were used in the flow cytometry experiments.
Gating strategy	The gating strategy is described in the aptamer selection section of the supplementary information provided and the gating strategy is exemplified in supplementary figure 17. In short, the singlet bead populations are identified by forward and side-scatter plot, and then the top ~0.1%-0.2% of the single bead population in the channel corresponding to the labeled glucose molecule is collected. No cells were used in this study, so there is no need to identify positive or negative cell staining populations.

☒ Tick this box to confirm that a figure exemplifying the gating strategy is provided in the Supplementary Information.
